# Targeted Therapy of Cancer Using Photodynamic Therapy in Combination with Multi-faceted Anti-Tumor Modalities

**DOI:** 10.3390/ph3051507

**Published:** 2010-05-14

**Authors:** Malini Olivo, Ramaswamy Bhuvaneswari, Sasidharan Swarnalatha Lucky, Nagamani Dendukuri, Patricia Soo-Ping Thong

**Affiliations:** 1 National Cancer Centre Singapore, 11 Hospital Drive, 169610, Singapore; E-Mails: dmsram@nccs.com.sg (R.B.); Lucky.S.S@nccs.com.sg (S.S.L.); Nagamani.P@nccs.com.sg (N.D.); nmstsp@nccs.com.sg (S-P.T); 2 Singapore Bioimaging Consortium, Biomedical Sciences Institutes, 11 Biopolis Way, #02-02 Helios, 138667, Singapore; 3 School of Physics, National University of Ireland, Galway, Ireland; 4 Department of Pharmacy, National University of Singapore, No. 18 Science Drive 4, Block S4, 117543, Singapore

**Keywords:** photodynamic therapy (PDT), targeted therapy, peptides, nanoparticles, vascular PDT, anti-angiogenesis, immune response

## Abstract

Photodynamic therapy (PDT) has emerged as one of the important therapeutic options in the management of cancer and other diseases. PDT involves a tumor-localized photosensitizer (PS), which when appropriately illuminated by visible light converts oxygen into cytotoxic reactive oxygen species (ROS), that attack key structural entities within the targeted cells, ultimately resulting in necrosis or apoptosis. Though PDT is a selective modality, it can be further enhanced by combining other targeted therapeutic strategies that include the use of synthetic peptides and nanoparticles for selective delivery of photosensitizers. Another potentially promising strategy is the application of targeted therapeutics that exploit a myriad of critical pathways involved in tumorigenesis and metastasis. Vascular disrupting agents that eradicate tumor vasculature during PDT and anti-angiogenic agents that targets specific molecular pathways and prevent the formation of new blood vessels are novel therapeutic approaches that have been shown to improve treatment outcome. In addition to the well-documented mechanisms of direct cell killing and damage to the tumor vasculature, PDT can also activate the body’s immune response against tumors. Numerous pre-clinical studies and clinical observations have demonstrated the immuno-stimulatory capability of PDT. Herein, we aim to integrate the most important findings with regard to the combination of PDT and other novel targeted therapy approaches, detailing its potential in cancer photomedicine.

## 1. Introduction

Photodynamic therapy (PDT) has emerged as a promising therapeutic modality for the treatment of oncological diseases [1,2]. It has the advantage of dual selectivity, due to the preferential localization of the photosensitizer by the malignant tissue and restriction of photoactivation to the tumor site due to localized light irradiation [3]. Furthermore it induces tumor vascular shutdown leading to thrombosis and haemorrhages, and local damage by recruitment of inflammatory and immune mediators [4]. Direct tumor destruction, tumor vasculature shutdown and anti-tumor immune response are three important cell death mechanisms in PDT. The combination of all three PDT mechanisms may lead to long-term tumor control via anti-tumor action against both the primary and metastatic tumors [1,2,5,6]. Therefore, PDT as a minimally invasive technique can be an attractive alternative to surgery or radiation with less morbidity and ability to preserve the anatomic and functional integrity of many organs such as the tongue, bladder or larynx with excellent cosmetic results. The evident advantage of PDT over other conventional cancer treatments such as chemotherapy and radiotherapy is its minimal side effects, selective targeting, no drug resistance and reduced toxicity that allows for repeated treatment [1].

Newer drugs that attain a higher degree of specificity by acting on specific molecular pathways have come to be known as targeted therapies. Chemotherapy and radiation kill predominantly proliferating cells and do not discriminate between tumor cells and normal host cells. Hence, there arises a need for targeted therapy to specifically target tumor cells in order to reduce normal tissue toxicity and side effects. The molecular identification of cancer antigens has opened new possibilities for the development of effective antibody therapy, immunotherapies and ligand-targeted therapy for cancer patients. Targeted therapy can also act as a complement to other existing cancer therapies such as chemotherapy, radiotherapy, immunotherapy and PDT [7]. 

This review will summarize the different molecular approaches that have been employed along with PDT to improve the targeted treatment of cancer. As most first-generation (haematoporphyrin derivative) and second-generation photosensitizers (phthalocyanines, benzoporphyrins, purpurins and chlorines etc.) used in PDT do not display significant tumor tissue selectivity there is a need for designing improved targeted delivery. Therefore, to enhance selectivity and overall efficacy of PDT, third-generation photosensitizers [8] are being designed and developed, by improving the existing photosensitizers, adding specific moieties and using delivery vehicles to specifically target these compounds [9]. Targeted-PDT by using these improved photosensitizers offers the added advantage of trafficking the photosensitizers across the cellular plasma membrane, resulting in intracellular accumulation of the photosensitizer. The use of nanoparticles as carriers of photosensitizers is a very promising approach as nano-materials can satisfy all the requirements for an ideal PDT agent [10]. In the nanoparticle-based approach, a photosensitizer is encapsulated or immobilized on the nanoparticle surface using covalent/noncovalent interactions. The advantage of this approach is the targeted delivery of the photosensitizer to the tumor site in a more selective manner and with low toxicity, rendering minimal damage to the normal tissues.

In recent years, the focus on the tumor vascular system has led to two new therapeutic approaches, vascular targeting and anti-angiogenesis. As abnormally enhanced neovascularization is a hallmark of tumor progression, it is important to evaluate vascular agents that exhibit properties that include high molecular weight and solubility in water/plasma to increase therapeutic selectivity. These properties would allow the photosensitizer to be confined in the vasculature for a longer period and would also reduce the problems of aggregation during administration [11]. Tumor angiogenesis is a highly complex multifactor process in which numerous molecular pathways are involved. Recent clinical studies have demonstrated that inhibiting angiogenesis after chemotherapy and radiotherapy is an attractive and valuable approach to cancer treatment [12]. In a similar way, anti-angiogenesis PDT has also shown promise as a cancer treatment modality as it disrupts supply of oxygen and nutrients to the tumor cells thus inhibiting tumor regrowth [13]. Apart from direct cell killing and damage to the tumor vasculature, PDT can also activate the body’s immune response against tumors [5,6]. Numerous pre-clinical and clinical observations studies have demonstrated the immuno-stimulatory capability of PDT. Therefore, PDT-activated anti-tumor immunity holds potential for the clinical treatment of both local and distant disease. 

We have organized this review into four sections: (1) synthetic peptides in targeted photodynamic therapy; (2) nanoparticle based drug delivery and targeting in PDT; (3) vascular and anti-angiogenesis targeted photodynamic therapy and (4) photodynamic therapy-mediated immune response. Within each section the different targeting approaches have been discussed. 

## 2. Synthetic Peptides in Targeted Photodynamic Therapy

Peptides are short biopolymers consisting of up to 50 amino acids in length, linked through peptide or amide bonds. Peptides generally do not possess a well-defined 3-dimensional or tertiary structure. Thus, their simple structure and smaller size make them good candidates for de novo synthesis as novel molecules. Moreover, they can be easily modified chemically and linked to spacers, water solubilising chelating moieties or radiolabels. Short synthetic peptides have the advantages of increased tissue permeability, rapid internalization capacity, effective receptor binding, rapid clearance and very mild antigenicity, which makes them ideal for the targeted delivery of photosensitizer through a receptor-mediated targeting approach.

**Figure 1:**
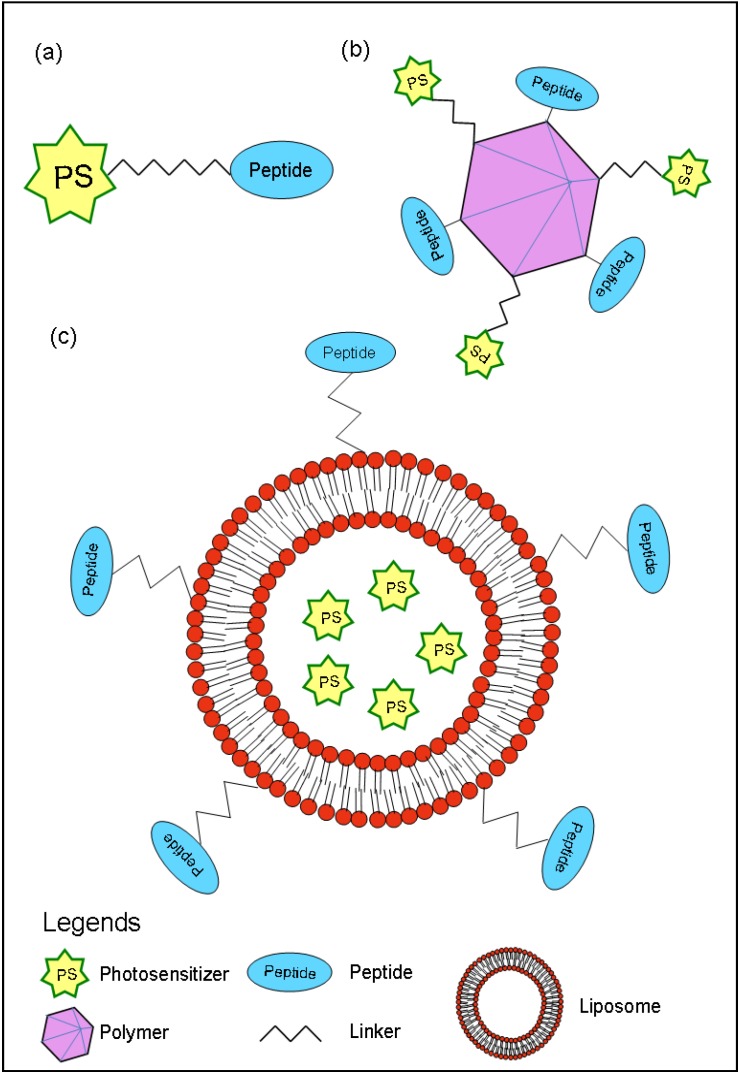
Design of photosensitizer conjugate: **(a)** peptides covalently attached to a photosensitizer via linker, **(b)** several peptides as well as photosensitizer molecules conjugated to a polymer scaffold, and **(c)** peptides covalently coupled to liposomes encapsulating PS.

A photosensitizer-peptide conjugate can be designed by direct covalent linkage of the peptide to the photosensitizers [14,15] or several peptides and photosensitizer molecules could be conjugated to a polymer scaffold [16]. Furthermore, liposomes encapsulating photosensitizers can be modified by coupling with peptides (Figure 1) [17,18].

### 2.1. Cellular-targeted PDT with synthetic peptides

A few studies have reported the use of charged peptides to increase cellular uptake. A polycationic or polyanionic photo-immunoconjugate was developed by bio-conjugating chlorin e6 (Ce6) with F(ab')2 fragment of the murine anti-ovarian cancer monoclonal antibody OC125 via a native poly-L-lysine linker and by a succinylated poly-L-lysine conjugate respectively. *In vitro* studies using these photo-immunoconjugate showed that the cationic variant was found to be more effective in killing ovarian cancer cells than the anionic counterpart due to its effective cellular internalisation through active endocytosis [19]. In addition, it was reported that the cationic photo-immunoconjugate undergo endosomal processing and were more likely to be degraded by lysosomal enzymes than the anionic photo-immunoconjugate and such a lysosomal degradation of photosensitizer conjugates can increase the phototoxicity of the photosensitizer by modifications in its photophysical state [20]. 

Synthetic peptides containing the Arg-Gly-Asp (RGD) motif have been used extensively to target alpha(v)beta(3) (αvβ3) integrin, and inhibit integrin-ligand interactions [21,22,23,24]. Integrins αvβ3 that have been implicated in misregulated angiogenesis as well as tumor growth and metastasis [25], are normally heavily expressed on tumor cells such as osteosarcomas, neuroblastomas and lung carcinomas and associated with actively proliferating endothelial cells. These cells bind to the components of the extracellular matrix (ECM) through their extracellular domains via a RGD motif. Solid-phase synthesis of four porphyrins bearing the RGD (H-Arg-Gly-Asp-OH) tripeptide targeted to αvβ3 integrin was reported by Chaleix *et al*. [21], out of which three displayed photodynamic activity on the K562 leukemia cell line to a degree comparable to that of native Photofrin II^®^. The cyclic dithiopentapeptide CRGDC with RGD motif containing S-S link has been found to adopt conformations showing increased affinity for integrins [26,27]. By substituting the disulfide bond with a C-C bond, a more enzymatically stable cyclic peptide with improved plasmatic residence time was reported [26]. Carboxy-glucosylporphyrins coupled to this cyclic peptide via a spacer arm showed the same efficiency for singlet oxygen production as hematoporphyrin under the same experimental conditions.

Several efforts have also been made to enhance delivery of the photosensitizer directly into the cell nucleus, as the presence of singlet oxygen in close proximity to the cancer cell DNA would dramatically increase the percentage of cell death whether by induction of apoptosis or necrosis. Several studies using the photosensitizer Ce6 linked to either a linear peptide (H-Ala-Pro-Pro-Lys-Lys-Lys-Arg-Leu-Val-Glu-Asp-Pro-OH) or branched peptides with eight identical peptide arms coding for two functional domains [(1) a pentalysine domain acting as a cytoplasmic transport sequence (CTS) and (2) the simian virus SV40 large T antigen nuclear localizing signal (NLS)], showed endocytosis and subsequent localization of these constructs within cell nuclei [28,29,30]. There was a 1.5 fold increase in nuclear localization of Ce6-branched peptide construct compared to Ce6-linear peptide variant in radiation-induced fibrosarcoma cells (RIF-1). Photodynamic activity of both conjugates against Chinese Hamster Ovary (CHO) cells was markedly increased. In another study, a SV40 large T antigen NLS (H-Pro-Lys-Lys-Lys-Arg-Lys-Val-OH) was directly bound to the boronated porphyrin [31]. This conjugate exhibited a non-covalent association with low density lipoprotein (LDL) receptors that is upregulated in many tumors including gliomas [32]. Thus, the porphyrin-NLS-LDL association could provide a selective entry pathway into malignant cells overexpressing the LDL receptors. 

Recently nano-sized peptide vesicles, with an average diameter of 120 nm, were explored as potential delivery system for photosensitizers. It was shown that recombinant amphiphilic oligopeptides with amino acid sequence Ac-Ala-Ala-Val-Val-Leu-Leu-Leu-Trp-Glu-Glu spontaneously assembled into nano-sized vesicles, which could be further stabilized by introducing multiple cysteine residues within the hydrophobic domain, thus allowing the formation of intermolecular disulfide bridges [33]. Poorly water-soluble phthalocyanines were quantitatively entrapped within the hydrophobic domains of these peptide vesicles. *In vitro* studies demonstrated that peptide vesicles bind to a range of cell lines resulting in effective cellular internalization, with clear co-localization of the photosensitizer and peptides inside endocytic compartments, via a receptor-independent route, presumably involving adsorptive macropinocytosis. Upon illumination, the phthalocyanine containing peptide vesicles showed an active photodynamic response towards the cells leading to effective cell killing [34].

Tsay *et al*. [35] used photosensitizer Rose Bengal linked to an *N*-hydroxysuccinimide (NHS) ester covalently coupled to lysine-terminated phytochelatin-related peptides (K-G-S-E-S-G-G-S-E-SG-Cha-C-C-Cha-C-C-Cha-C-C-Cha-Cmd) and then coated green or red emitting CdSe/CdS/ZnS QDs with the modified peptides. Similarly they also synthesised QDs coated with peptide-Ce6 conjugates. There was a dramatic increase of fluorescence of both peptide-Rose Bengal-green QD conjugate as well as peptide-Ce6-red QD conjugate as a result of Förster (fluorescence) resonance energy transfer (FRET), in comparison with photosensitizers alone at the same concentration. These conjugates could efficiently generate singlet oxygen for PDT either through direct excitation of photosensitizers or through indirect excitation through FRET from the nanocrystals to photosensitizers. Singlet oxygen quantum yields as high as 0.31 was produced by peptide-Ce6-QD conjugate by direct excitation of the sensitizer using 532 nm excitation wavelengths [35]. Further *in vivo* studies demonstrating targeting, imaging and PDT in live animals are yet to be carried out. Other quantam dot-PS complexes have been described in the nanoparticles section.

Similar studies with commercially available PS, protoporphyrin IX (PpIX) conjugated with cyclic peptide, cRGDfK (Arg-Gly-Asp-Phe-Lys) showed good photodynamic activity *in vitro* and *in vivo*, using αvβ3-positive human cervical cancer SiHa cell line and a mouse CaNT mammary carcinoma model respectively [24]. Pharmacokinetic analysis of PpIX-cRGDfK conjugate treated mice shows significant retention and accumulation of PS in tumor tissue with higher tumor to normal tissue ratios than the free PS, however the overall *in vivo* treatment efficacy did not show much improvement due to the possible differences in the target environment or in the subcellular localisation of the compounds. Thus careful selection of the type of PS, treatment conditions and compound metabolism is vital for the exploitation of this technology to its full potential. *In vivo* studies using cationic photo-immunoconjugate demonstrated superior tumor selectivity and best tumor to normal ratio when delivered intraperitoneally to a nude mouse xenograft model of human epithelial ovarian cancer [36]. 

In order to improve the therapeutic efficacy of PDT using the PS, benzoporphyrin derivative monoacid ring A (BPD-MA), Ichikawa *et al*. [37] synthesized polyethylene glycol (PEG)-modified liposomes encapsulating BPD-MA (PEG-Lip-BPD-MA). The PEG-coating is known to enhance the passive accumulation of liposomal drugs in tumor tissues of tumor-bearing animals. Thus, though the PEG moiety led to the enhanced accumulation of BPD-MA in Meth-A-sarcoma tumor tissues up to 4 folds compared to BPD-MA delivered with non-modified liposomes at 3 h after injection, it decreased the suitability of the drug for PDT when laser irradiation was performed. To improve the bioavailability and intracellular internalisation of PEG-Lip-BPD-MA it was modified with the inclusion of an APRPG (H-Ala-Pro-Arg-Pro-Gly-OH) pentapeptide specific to angiogenic endothelial cells [17,38]. Though the degree of accumulation of the APRPG peptide modified PEG-Lip-BPD-MA was similar to the PEG-Lip BPD-MA, this treatment strongly suppressed tumor growth after laser irradiation and significantly damaged vasculature in the dorsal air sac of the angiogenesis model.

### 2.2. Vascular-targeted PDT with synthetic peptides

Development of peptide-PS conjugates targeting tumor vasculature is another approach of achieving targeted PDT by potentiating the vascular effects of PDT, which is thought to play a major role in tumor eradication. Vascular endothelial growth factor (VEGF) plays an important role in the process of angiogenesis. It stimulates vascular endothelial cell growth, facilitates survival of existing vessels and the proliferation and migration of endothelial cells [39]. VEGF binds to kinase receptors such as VEGFR-1 and -2, expressed on vascular endothelial cells; as well as neuropilins, NRP-1 and -2, expressed both on vascular endothelial cells and some tumor cells [40]. A photosensitizer 5-(4-carboxyphenyl)-10,15,20-triphenyl-chlorin (TPC), was coupled to a VEGF receptor-specific heptapeptide, H-Ala-Thr-Trp-Leu-Pro-Pro-Arg-OH (ATWLPPR), via a spacer (6-aminohexanoic acid, Ahx) [41]. TPC–Ahx–ATWLPPR bound exclusively to NRP-1, accumulated up to 25-fold more in HUVEC compared to free TPC over a 24 h period, thus exhibiting 10.4-fold enhanced *in vitro* photodynamic activity compared to free TPC. *In vivo* experiments using U87 human malignant glioma cells xenografted nude mice revealed significant tumor to normal ratios as early as 1 h after intravenous injection of TPC–Ahx–ATWLPPR. Taken together, TPC–Ahx–ATWLPPR is a much more potent PS *in vitro* than TPC, in NRP-1-expressing cells. 

Frochot *et al*. compared the *in vitro* selectivity and photodynamic activity of the PS (5-(4-carboxyphenyl)-10,15,20-triphenylchlorin or porphyrin coupled with linear RGD triad or cyclic RGD motif in αvβ3-positive human umbilical vein endothelial cells (HUVEC) and αvβ3-negative murine mammary carcinoma cells (EMT-6) [23]. Their results showed that RGD-containing linear or cyclic peptide targeted tetraphenylchlorin were incorporated in HUVEC up to 98- and 80-fold more, respectively, than the unconjugated PS over 24 h. However, a non-specific cellular uptake by EMT-6 lacking αvβ3 receptors was also observed. Survival measurements clearly demonstrated superior sensitivity of HUVEC to peptide conjugate-mediated PDT than the unconjugated PS, due to its higher cellular uptake. Thus, a PS with linear or cyclic RGD motif not only has the potential to target tumor endothelial cells for efficient PDT, but the peptidic moiety also introduces a hydrophilic/hydrophobic balance thus exhibiting excellent water solubility and weak tendency to form aggregates.

Thus, targeted-PDT via receptor specific synthetic peptides has made reasonable progress in the recent years to achieve a certain degree of selectivity by site-specifically confining the PS, and thereby increasing the efficacy of PDT.

## 3. Nanoparticle-Based Drug Delivery and Targeting in PDT

Since most of the photosensitizers employed in PDT are hydrophobic and show poor water solubility. This makes it difficult to administer these drugs for *in vivo* applications [42]. In addition to this, the low selectivity of the drug to the tumor site makes it difficult to use the PS in a clinical set up. In this context, nanoparticle based approaches have been utilized in selective introduction of the PS to cancerous cells. In this emerging mode of delivery, a PS is encapsulated or immobilized on the nanoparticle surface using covalent/noncovalent interactions. The advantage of this approach is that the PS is delivered to the tumor site in a more selective manner with low toxicity, rendering minimal damage to the normal tissues [10]. In this approach, nanoparticles can be used as drug carriers where the drug is either dissolved in the matrices or adsorbed on the surface. This approach has been gaining attention in the recent years because of the tunability of size, surface characteristics and high drug loading capability of the nanoparticles [43]. Other kinds of PS-loaded nanoparticles, such as fullerene based, liposome based and dendrimer based nanoparticles that are reviewed by Toru Oba [44] are not discussed in this section. Nanoparticles can be biodegradable polymer based, such as chitosan [45] and poly lactic acid derived [46] or inorganic particles such as silica [47] or gold [48] and quantam dots [49].

### 3.1. Cellular-targeted nanotherapy

Amongst the biodegradable polymers, chitosan based nanoparticles (CNPs) have been developed as nanodrug carriers for the tumor specific delivery of PpIX encapsulated with glycol chitosan 5b-cholanic acid (PpIX-NP) for PDT. These nanoparticles showed better tumor specificity and improved therapeutic efficacy compared to the free PpIX [45]. Advantages of these CNPs are that they can incorporate the hydrophobic drugs to their hydrophobic inner core and cells could readily take up these water soluble CNPs to the cytoplasm. 

Hone *et al*. reported the use of phthalocyanine stabilized gold nanoparticles as delivery vehicles based on metallic nanoparticles [50]. They enhanced the solubility by utilization of tetra octyl ammonium bromide as a phase transfer agent enhanced the solubility of the surface bound hydrophobic phthalocyanine in polar solvents. These three component nanoparticles were found to be better in synstemic injection of the phthalocyanine by generating singlet oxygen with enhanced quantum yield compared to the free phthalocyanine. The follow up work by the same group [48], described the incubation of these nanoparticle conjugates with HeLa cells, and found that they were taken up by the cells. Irradiation of the cells with nanoparticle-phthalocyanine conjugates induced cell death through the production of singlet oxygen. In a recent approach, pegylated gold nanoparticle silicon phthalocyanine (AuNp-Pc4) conjugates have been developed as efficient drug vectors for PDT drug delivery. AuNp-Pc4 conjugates are water-soluble, biocompatible and showed an improvement in the delivery of the PS to the tumor site compared to conventional administration of photosensitizers. In another approach Chen *et al*. [51] utilized luminescent nanoparticles for the delivery of PSs like porphyrins. Upon exposure to x-rays, these particles emit scintillation that activates the PS to generate singlet oxygen, without the need for an external light source.

Studies have shown that quantum-dot (QD)-PS complexes have clear advantages over PS alone due to the large absorbance cross-section, tunable optical properties and imaging capabilities, and a variety of schemes available to bioconjugate QDs [49,52,53]. The conjugation of QD to PS provides flexibility to utilize variable excitation wavelength to activate the PS molecule since the QDs exhibit broad absorption spectra. However, most of these complexes are not soluble in water, and therefore are not optimal for biological environments. Peptide-coated QDs form extremely stable conjugates with PSs, which may be used as targeted multifunctional probes for live cell targeting, imaging and PDT.

In addition to targeting, nanoparticle based approach can have advantages such as, increasing light penetration which is a crucial for effective PDT. To overcome this limitation nanoparticles for two photon PDT [54] have been developed. Two-photon absorption induced excitation is very promising as it uses two photons of lower energy (red shift, IR region) to produce an excitation that would otherwise be produced by the absorption of single photon of high energy (blue shift). Hence this method avoids wasteful absorption and facilitates deeper penetration of light into tumor tissues compared to the single photon PDT drugs. This feature has been used in the treatment of gliomas as these tissues need higher penetration power. The major drawback of single photon PDT drugs are that they are excited by visible light, which has limited penetration depth due to the restrictive tissue transparency window for target cells that are located deep inside living tissues. Photons in this spectral region do not penetrate deep enough, thus making single-photon PDT less effective for the treatment of gliomas. In another approach, up-conversion phosphors for the ceramic-based nanoparticles have been utilized by Ungun *et al*. [55] for PDT. Up conversion phosphors are ceramic materials with a rare earth atom in the crystalline matrix. These absorb light in the near IR region and up convert to emit in the visible region. Advantages are that the power required to excite these particles is 10^7^ times less than the intensities needed for two photon excitation of conventional organic dyes and they are resistant to photo bleaching. In a similar approach Zhang *et al*. [56] have described NaYF_4_:Yb3+, Eu3+ nanoparticles as photon up converting nanoparticles (PUNPs) and these particles were recognized as being some of the most efficient photon upconverting phosphors [57].

In summary, nanoparticles offer a versatile platform for PDT drug delivery by targeting and with additional advantages such as enhanced light penetration. This section describes the various nanoparticle based drug deliveries in targeted PDT using biodegradable and non-biodegradable nanoparticles. Upconverting nanoparticles provide an advantage by converting low energy radiation to high-energy emission thereby facilitating the use of these particles for PDT in deep-seated tumors. Gold nanoparticles with PEG shielding provide stable particles that can have longer circulation times in the body. Thus by choosing suitable nanoparticles, this approach could be used not only in improving the efficacy of PDT but also extended to enhance diagnosis and imaging.

## 4. Vascular and Anti-Angiogenesis Targeted Photodynamic Therapy

Destruction of functional vasculature is essential for efficient tumor eradication by PDT. Abnormally enhanced neovascularization is a hallmark of tumor progression and this has led to the concept of targeting the tumor vasculature as a therapeutic strategy. Therefore, either selectively targeting existing blood vessels and/or inhibiting the formation of new blood vessels can improve treatment efficacy [58]. In recent years, vascular-targeted PDT has received considerable attention as a promising strategy in cancer treatment and vascular-targeting PSs such as TOOKAD and verteporfin are currently in clinical development [59,60]. In this section, we will discuss the use of vascular disrupting and anti-angiogenic agents that have significantly contributed to the final outcome of PDT.

### 4.1. Vascular-targeted PDT

**Figure 2:**
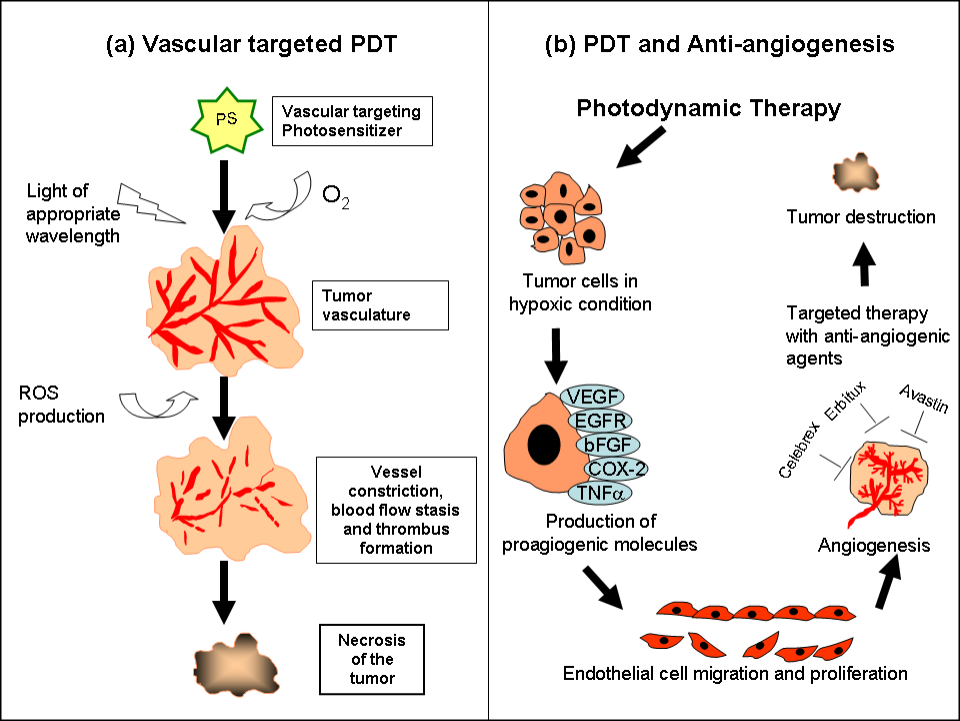
**(a)** Vascular-targeting PDT (VTP) is performed when the PS is confined within the tumor vasculature. Following VTP, the sensitive sites within the microvasculature are damaged thus causing vessel constriction, blood flow stasis and thrombus formation that finally leads to tumor destruction. **(b)** PDT induced oxidative stress causes hypoxia within the tumor tissue that triggers the release of angiogenic growth factors that include VEGF, COX-2 and EGFR. These pro-angiogenic factors facilitate endothelial cell proliferation and migration thus initiating angiogenesis and tumor growth. Administration of angiogenic inhibitors that specifically target these growth factors in combination with PDT has shown to be effective in suppressing angiogenesis and preventing tumor regrowth.

Vascular-targeting PDT (VTP) is characterized by a short drug to light interval (DLI), typically 0 to 30 minutes, when the PS is confined within the tumor vasculature. Following PS accumulation and irradiation, damage to sensitive sites within the microvasculature causes increases in vascular permeability and vessel constriction thus resulting in tumor destruction by vascular collapse, blood flow stasis and tissue hemorrhages (Figure 2a) [61]. Vascular targeting can be passive when the injected PS is largely confined in the blood vessels and reaches peak plasma concentration, thus providing a therapeutic window for vascular treatment. Active vascular targeting PDT, on the other hand is the delivery of photosensitizing compounds that selectively accumulates in the targeted neovascular components thus eliciting a preferential vascular response [62].

Vascular effects of PDT can differ greatly based on the different PSs and the drug-light interval administered. Tumor cell death after photoradiation with hematoporphyrin derivatives is caused by vasoconstriction and complete stasis that occurs secondary to destruction of the microvasculature [63]. Morphological changes after hematoporphyrin derivative PDT led to the absence of the endocapillary layer and mitochondrial degeneration, thus enhancing tumor destruction [64]. On the other hand, VTP with verteporfin is known to causes a dose- and time-dependent increase in vascular permeability and decrease in blood perfusion. The correlation between the timing for vascular damage and cure implies that blood flow stasis plays a significant role in PDT-induced tumor destruction. Verteporfin VTP has also been shown to permeabilize blood vessels through the formation of endothelial intercellular gaps thus triggering the loss of endothelial barrier function that leads to tumor vascular shutdown [65,66]. Photofrin PDT has been shown to induce changes in vessel constriction, vessel leakage and thrombus formation [67] and PDT with higher Photofrin dosage causes vessel constriction and changes in permeability during PDT [68]. PDT with certain phthalocyanine derivatives leads to vascular leakage [69] and mono-L-aspartyl chlorin e6 (NPe6) PDT has shown to cause blood flow stasis.

Combretastatin A4 phosphate (CA4P), a blood flow inhibitor has also exhibited potential as a vascular-targeting PDT agent by significantly increasing endothelial cell apoptosis thus enhancing the therapeutic effect [70]. VPT with BF2-chelated Tetraaryl-Azadipyrromethene agents, an emerging class of non-porphyrin PDT agent, in an *in vivo* mammary tumor model showed a decrease in tumor vascular perfusion and concomitant reduction in tumor metabolism over time after treatment [71]. Another potent vascular targeting agent, 5,6-Dimethylxanthenone-4-acetic acid (DMXAA) has been shown to be effective against a variety of experimental rodent tumors and xenografts and is currently undergoing clinical evaluation. In an *in vivo* Colon-26 tumor model, it was demonstrated that the combination of PDT and low-dose DMXAA causes selective tumor vascular response by increasing vessel permeability, leading to cell damage and loss of blood flow and thus resulting in >70% cure rate [72]. PDT with a novel PS MV6401 in a mammary tumor (MCaIV) caused dose-dependent blood flow stasis and long-term vascular shutdown, caused by thrombus formation [73]. Hypericin, a perylenequinone, has shown potential as a vascular targeting agent by improving cure rates in radiation-induced fibrosarcoma-1 solid tumors when short drug-light interval PDT was administered [74]. VTP with WST11, a water-soluble palladium-bacteriochlorophyll derivative, was able to produce long-lasting systemic anti-tumor immunity involving both cellular and humoral components, which could enhance host anti-tumor immunity for better disease control. 

### 4.2. Cellular-targeted PDT and anti-angiogenesis therapy

Although VTP induced microvascular damage causes extensive central tumor necrosis contributing to greater tumor response, tumor vessels or cells can re-grow from the peripheral rim after treatment. Hypoxia induced inflammatory responses in PDT is also known to diminish the treatment efficacy by promoting signaling cascades such as VEGF, cyclooxygenase-2 (COX-2), metalloproteinases (MMPs) and other cytokines, creating enhanced environment for tumor recurrence (Figure 2b) [75]. Consequently, efforts are underway to optimize PDT protocols for improved efficacy by targeting the pro-angiogenic growth factors with specific angiogenesis inhibitors. Anti-angiogenic agents may augment the activity of PDT by inhibiting its counterproductive upregulation of pro-angiogenic growth factors. 

VEGF is the one of the most potent and specific regulators of angiogenesis that triggers tumor regrowth by promoting proliferation, migration and tube formation of endothelial cells. Targeting VEGF with specific inhibitors in combination with chemotherapy, radiotherapy and PDT has shown potential to be an attractive and effective approach to cancer treatment. Combination treatment of PDT with a humanized monoclonal antibody, Avastin that targets and blocks VEGF exhibited long-time responsiveness in treated Kaposi’s sarcoma tumor [76]. Similarly, our group tested the efficacy of Avastin along with PDT in a bladder tumor xenograft model. The results demonstrated that the targeted therapy using Avastin along with PDT improved tumor responsiveness by inhibiting not only VEGF expression but also other angiogenic proteins such as angiogenin, basic fibroblast growth factor (bFGF), epidermal growth factor (EGF), interleukin-6 (IL-6) and interleukin-8 (IL-8) [77]. PDT followed by administration of a VEGF targeted anti-angiogenic agent, TNP-470, abolished the increase in VEGF expression and reduced local tumor growth in an orthotopic model of prostate cancer (LNCaP) [78]. Reduced tumor volume and prolonged survival time of glioma-implanted animals was observed by targeting VEGF receptors VEGFR-1 and VEGFR-2 by anti-angiogenic monoclonal antibodies MF1 and DC101 along with PDT [63]. Our earlier studies using hypericin-PDT demonstrated that predominantly vascular targeting short DLI PDT attenuated the expression of angiogenic growth factors compared to cellular targeting long DLI PDT [79].

Receptor tyrosine kinase (RTK) pathways are critically involved in the development and progression of cancers [80]. Therefore, signaling pathways controlled by tyrosine kinases offer unique opportunities for pharmacological intervention. Studies on the use of synthetic RTK inhibitors SU5416 and SU6668 along with hypericin PDT demonstrated extended survival of tumor-bearing host mice [81]. In the same way, another set of RTK targeted inhibitors PD166285 and PD173074 when combined with hexylether pyropheophorbide-PDT significantly decreased tumor regrowth and displayed potent anti-angiogenic and anti-tumor activity in a transplanted murine mammary 16c tumor model [82]. 

Cyclooxygenase-2 enzyme is an important mediator of angiogenesis and is expressed in a wide range of preneoplastic and malignant conditions. Targeting COX-2 using celecoxib and NS-398 with PDT caused increased *in vitro* apoptosis and attenuated *in vivo* inflammatory and angiogenic factors leading to enhanced treatment efficacy. Similarly, COX-2 targeted therapy with celecoxib or NS-398 and PDT exhibited significant improvement in long-term tumor free survival [83]. Another COX-2 inhibitor, nimesulide, potentiated anti-tumor effects of Photofrin PDT leading to complete tumor response in an *in vivo* colon adenocarcinoma tumor model [84]. In our earlier study, we reported downregulation of COX-2, HIF-1α and VEGF A isomers 165 and 121 when celebrex was administered 6 h post-PDT in an *in vivo* nasopharyngeal tumor model [85]. Targeting COX-2 using NS-398 and Npe6-PDT significantly decreased the weight of colon-38 tumor xenografts [86]. Administration of p38 MAPK inhibitor PD169316 abrogated COX-2 expression in PDT-treated cells. PDT in combination with pyridinyl imidazole, inhibitors of p38 MAPK, has also been reported to improve the therapeutic efficacy of PDT by blocking COX-2 upregulation [87].

Elevated levels of the epidermal growth factor receptor (EGFR), a growth-factor-receptor tyrosine kinase, has been identified as a common component of multiple cancer types and appear to promote growth of tumors [88]. Several studies have reported the targeting of EGFR along with PDT as a promising therapeutic approach in treating tumors. Well-differentiated NPC HK-1 cells when subjected to PDT with 1 μM of Zn-BC-AM and in the presence of EGFR inhibitor AG1478 increased the inhibition of EGFR/PI3K/Akt and EGFR/MEK/ERK signaling pathways [89]. Another report indicated EGF-stimulated phosphorylation on Y1068 as the most sensitive target on EGFR to PDT with amphiphilic PSs [90]. To test the potential of immunophotodiagnosis as a feedback modality for therapeutic intervention, experiments have been conducted with the anti-EGFR MAb (C225) conjugated to Ce6 followed by illumination to reduce expression of the EGFR via photodynamic effect. Subsequent immunophotodiagnosis showed that this treatment led to a significant reduction in fluorescence in the carcinogen-treated cheek pouch compared with nonilluminated areas, thus suggesting EGFR as an appropriate target in head and neck cancer [91]. Similarly, a mechanistically nonoverlapping combination modality consisting of EGFR inhibition with C225 and BPD-PDT was known to be well tolerated, effective, and synergistic in mice [92]. Silicon phthalocyanine (Pc4) mediated-PDT resulted in a time-dependent inhibition of protein expression of EGFR and tyrosine phosphorylation of EGFR by inducing apoptosis and improving treatment efficiency [93]. In our anti-angiogenesis study, we demonstrated that combination of EGFR targeted antibody, Erbitux with PDT strongly inhibited tumor growth in the bladder tumor xenograft model. Downregulation of EGFR, increased apoptosis, dephosphorylation of ErbB4 at tyrosine 1284 site and downregulation of EGFR target genes cyclin D1 and c-myc played a major role in tumor inhibition [13].

In conclusion, targeting tumor vasculature with vascular and anti-angiogenic agents along with PDT is a promising treatment modality that can destroy tumor cells. An essential prerequisite to target vasculature is a photophysically defined PS localizing in the intravascular space and a specifically targeted angiogenic inhibitor with minimal toxicity.

## 5. Photodynamic Therapy-Mediated Immune Response

In addition to direct tumor cell killing and causing damage to tumor vasculature as discussed in the preceding sections, PDT has been shown to activate an anti-tumor immune response (reviewed in [5] and [6]). Thus a local treatment of tumors may lead to a systemic immune response against tumors outside of the treatment field. In recent years, there has been growing interest on this third aspect of PDT-mediated tumor destruction, as a combination of all three PDT mechanisms may lead to longer-term tumor control via anti-tumor action against both the primary and metastatic tumors [1,2,5,6]. PDT-activated immunity may therefore play an important role in determining the long-term treatment outcome. In this section, we will provide a brief overview of the PDT-mediated immune response as demonstrated in both pre-clinical and clinical studies.

### 5.1. Pre-clinical studies

Numerous pre-clinical studies have demonstrated the immuno-stimulatory capability of PDT and only a few will be highlighted here to illustrate the point. A study comparing the long-term effects of PDT between immuno-competent and immuno-deficient mice showed that the activity of host lymphoid populations was essential in preventing recurrence of tumors following PDT and that PDT-induced immune response contributed to the overall cure rate [94]. PDT has also been shown to result in the generation of tumor-sensitized immune cells that assert immune memory and that can be recovered from distant lymphoid sites even after long intervals following PDT [95]. In another study, antigen-presenting cells (APCs) isolated from PDT-treated mice were reported to exhibit an enhanced ability to stimulate T cell proliferation and T cell secretion of interferon-γ [96]. The results suggested that APC activity is increased following PDT. A more recent study showed that local PDT of tumors resulted in an enhanced anti-tumor immune memory, leading to CD8+ T cell control of tumors that were outside of the treatment field [97].

### 5.2. PDT-generated anti-tumor vaccines

In addition to the role of PDT in stimulating the host immune response, there’s also interest in the potential of using PDT to generate anti-tumor vaccines. Gollnick *et al* reported that PDT-generated tumor cell lysates are strongly immunogenic and are effective anti-tumor vaccines [98]. Likewise, Korbelik *et al* successfully generated vaccines using PDT on both squamous cell carcinoma (SCC) cells *in vitro* and *ex vivo* SCC tumors. Such approaches point the way to PDT-generated vaccines targeting specific antigens of the patient’s tumor, and hence are tailored to individual patients [99]. A Phase I clinical trial is currently underway to study the clinical efficacy of PDT-generated vaccines to activate immune responses to melanoma [6]. In this study, autologous vaccines generated by PDT treatment of surgically removed melanomas will be used to treat patients with advanced stage II in transit melanoma.

### 5.3. Clinical studies

Significantly, PDT-activated anti-tumor immunity has also been linked to clinical outcome. It has been observed that vulva intraepithelial neoplasia (VIN) non-responders to PDT were more likely to show HLA class I loss compared to responders. A significant increase in the CD8 infiltrate was also observed in post-PDT responders compared to non-responders, suggesting that cell-mediated immunity may play an important role in the response of VIN to PDT [100].

We have also reported clinical observation of a PDT-activated immune response against distant untreated angiosarcoma lesions [101]. Following PDT of lesions in the head and neck region, new lesions appeared on both upper limbs, whereupon the main cluster of lesions on the right upper limb were treated with PDT. The untreated lesions on the right and left upper limbs underwent spontaneous remission 2 months and 4 months after PDT respectively. To the best of our knowledge, this was the first clinical case in which untreated distant tumors were observed to regress following PDT. These clinical observations were supported by results of immunohistochemical studies carried out on the same patient, when lesions were observed to recur at the head and neck region. PDT was repeated on only one lesion on the right occiput. Biopsy of the untreated left occiput at 48 h after PDT showed a predominantly CD4+, CD8- T lymphocyte infiltrate while repeat biopsy of the same site one month post-PDT showed a predominantly CD4-, CD8+ T lymphocyte infiltrate [101]. Five and a half years after the first PDT, this patient remains disease-free.

Our clinical observations led us to hypothesize that following PDT, immunogenic peptides generated by dying cells are presented by APCs, resulting in a cell-mediated immune response against untreated tumors via the activation and proliferation of cytotoxic T-cell clones [95]. Our hypothesis is in agreement with the pre-clinical observations that PDT increased APC activity and that local PDT of tumors can lead to induction of CD8+ T cell-mediated control of distant tumors [96,97].

In a recent clinical study, it was reported that basal cell carcinoma (BCC) patients who underwent PDT showed an increased systemic immune response to a BCC associated tumor antigen compared to patients who underwent surgical removal of the tumors [102]. The results demonstrate that local PDT can enhance systemic anti-tumor immunity.

Since PDT works via the interaction between a photosensitizing drug, PS-activating light and tissue oxygen, the outcome following PDT is dependent on the parameters used during the treatment. Parameters that can affect the outcome include the intracellular distribution of the PS [103], the PS and light doses [104], the time interval between PS administration and light activation [105], the light fluence rate [106,107,108,109] and more recently, the duration of light treatment [110]. 

The fluence rate (the rate at which the PS-activating light is delivered) is known to be one of the key parameters in PDT that can be varied to modulate the PDT outcome. Specifically, low fluence rate has been shown to work via different ways to elicit a better PDT response. For example, the fluence rate has been shown to affect the intra-tumoral inflammatory response, with a strong inflammatory response significantly contributing to tumor control, especially when the PDT regimen is suboptimal [106]. Fluence rate has also been reported to affect tumor response, with low fluence rate promoting tumor control and high fluence rate counteracting tumor control, through its oxygenation modulating properties and possibly other mechanisms [107]. A recent study demonstrates that low fluence rate results in reduced intra-tumor heterogeneity in hypoxic, vascular and cytotoxic responses, thereby offering better outcome [109].

Further to our clinical observation of PDT-activated anti-tumor immunity, we have posited the fluence rate to be a modulator of the PDT-mediated immune response [108]. High dose PDT carried out at a high fluence rate resulted in local control of the disease but no immune response was observed, whereas PDT with a lower fluence and fluence rate resulted in better outcome, including activation of the body’s immune response against distant untreated lesions.

Our clinical observations are in agreement with pre-clinical and clinical studies showing that enhanced PDT-activated immunity correlates with both lower fluence and lower fluence rate [102], [111]. The study by Kousis *et al* showed that induction of anti-tumor immunity following PDT is dependent on infiltration of neutrophils (pro-inflammatory cells) into the treated tumor bed, which in turn is dependent on the fluence and fluence rate [111]. The clinical study by Kabingu *et al* showed that treatment of BCC lesions with lower light doses resulted in greater enhancement of immune recognition of the BCC associated antigen, Hip1 [102].

In summary, PDT-activated anti-tumor immunity holds potential for the clinical treatment of both local and distant disease. Results from both pre-clinical and clinical studies show that it might be possible to modulate the PDT-mediated immune response via optimisation of the fluence rate or fluence rate in combination with the light fluence. The careful design of a PDT treatment regime leading to enhanced anti-tumor immunity thus offers the prospect of longer-term clinical tumor control.

## 6. Conclusions

This review highlights the recent diverse strategies to achieve targeted PDT by developing improved PSs or by combining PDT with vascular and anti-angiogenic agents, as well as by modulating PDT-mediated immune response. Careful selection of the PS, rational design and synthesis of the targeting moieties against specific receptors, design and development of efficient delivery vehicles and strategic incorporation of the PS into the delivery vehicles are critical for further improving and creating a PDT paradigm based on these targeted approaches. These targeted approaches along with appropriate combination of vascular and anti-angiogenic agents and tactical fine-tuning of light dosimetry to elicit an effective immune response could bring PDT to the forefront of cancer therapeutics.
